# Report of the Diseases of Edinburgh for October, 1809

**Published:** 1809-12

**Authors:** John Roberton


### *Report of the Diseases of Edinburgh for October,* 1809.

By John Roberton, M. D.

The weather, during the whole of this month, as is indeed not unusual after the proper period has gone by, has been delightful. Thus our February and March are often more agreeable than the two months following; and October, as in the present season, is not uncommonly more pleasant than two months previous to it. This irregularity of seasons is often productive of serious consequences, especially to the infirm. Indeed, to all ranks it is prejudicial, owing, in a great measure, to the prevalent custom of dressing, particularly among ladies, rather according to the appearance of the day than to the advancement of the season. I am convinced, that in a climate such as this, where at best the summer is so short, and even then so often interrupted by unfavourable circumstances, there can be but few apologies adduced in favour of too thin dress, and many diseases of the most fatal kind may easily have their origin traced to no other cause. This practice, I have no hesitation in deprecating, even during that time of the year when we are most likely to have good weather; but at such an advanced state of the season as this, when, whatever the days (now much shortened) may be, the nights must be cold and disagreeable, such habits cannot be too much condemned. The mornings and evenings during the whole month were frosty.

The barometer, soon after the commencement of the month, rose considerably, and continued high throughout the whole of it.

The thermometer also stood rather high considering the advanced period of the year. It, however, uniformly fell considerably every night after sun-set, and continued low till the forenoon of the following day.

The most prevalent diseases of last month still continue. The most common and most severe, however, are diarrhœa and catarrh. Small-pox and ophthalmia have been added to the list.

It is a pity that small-pox should still be allowed to prevail in the list of our diseases, when its antidote the cow-pox is so conspicuously serviceable.

Ophthalmia has appeared in various parts of the city; in some instances very severely; so much so, indeed, as almost completely to deprive the patient of sight. In general, however, it has not been very severe. As I formerly had occasion to mention, in a general way, (and it cannot not be too often stated) the treatment of this disease is almost always conducted, in every variety of constitution, nearly on similar principles; as by the application of cold in various ways to the affected eyes; a wash for them composed of Goulard's extract of lead, or some one or other of the vitriolic preparations; the division of the blood vessels running over the ball of the eye, and the administration of brisk purgatives. Instead of this, I scarcely recollect of any disease in which the discriminating powers of the medical attendant are more necessary than in this; as, owing to the particular habit of body in which it appears, the means by which it may be best removed are very opposite to each other. In scrofulous patients, for instance, scarcely any one of the above enumerated applications will do any good; but, on the contrary, often occasion much pain and unnecessary distress. I may, indeed, except from this, the occasional administration of purgative medicines when the bowels are constipated, and the division of the blood vessels running over the affected eye, especially when they are greatly enlarged, and not likely to be destroyed by milder means; but, as stated, in such patients, all the other applications of a cooling kind are decidedly bad. Indeed, I have no hesitation in asserting, that the same reasoning may be applied to habits much debilitated by age, disease, or improper living. On the contrary, the applications of most general use for the removal of ophthalmia in scrofulous patients, are of a stimulating nature, and immediately applied to the affected eye. Such are strong ardent spirits, or laudanum, blown twice a day through a goose quill into the eye, while the eye-lids are held open by another person, or a liniment composed of the red oxyd of mercury and simple ointment, introduced within the eye-lids once or twice a day.

When we have ascertained the peculiarity of the patient's constitution, whether of a scrofulous habit, or whether no such disease can be traced in his system, these states will at once point out the treatment most likely to be successful in the removal of the affection.

